# The inverted pattern of circulating miR-221-3p and miR-222-3p associated with isolated low HDL-C phenotype

**DOI:** 10.1186/s12944-018-0842-1

**Published:** 2018-08-16

**Authors:** Yuntao Zhou, Mengdi Liu, Jinrong Li, Bing Wu, Wei Tian, Lu Shi, Jing Zhang, Zening Sun

**Affiliations:** Tangshan Key Laboratory of Clinical Molecular Diagnosis and Treatment, Tangshan Gongren Hospital, No. 27 Wenhua Road, Tangshan, Hebei 063000 People’s Republic of China

**Keywords:** Isolated risk phenotype, Circulating microRNAs, Cardiovascular disease, Clinical translation

## Abstract

**Background:**

We investigated the baseline characterization of cardiovascular disease (CVD)-derived circulating miR-221-3p/222-3p in isolated low HDL-C phenotype (ILHP) to enhance our understanding on their molecular pathological pattern prior to disease onset.

**Methods:**

We screened 174 asymptomatic subjects with isolated low HDL-C phenotype (*n* = 88) and normal lipid phenotype (*n* = 86), and detected circulating levels of CVD-derived circulating miR-221-3p/222-3p using TaqMan miRNA Real-time PCR detection system.

**Results:**

We found the inverted pattern of decreased circulating miR-221-3p (0.415 [0.249, 1.004] vs 0.658 [0.347, 1.534], *p* = 0.002) versus increased miR-222-3p levels (0.379 [0.101, 0.701] vs 0.156 [0.043, 0.407], *p* < 0.001) in ILHP. The baseline levels of circulating miR-221-3p and miR-222-3p are correlated with serum HDL-C levels (miR-221-3p: *r* = 0.306, *p* < 0.001; miR-222-3p: *r* = − 0.201, *p* = 0.008). Gender-based analysis showed female-specific elevation of circulating miR-221-3p in asymptomatic individual. Multiple logistic regression analysis showed that circulating miR-222-3p is robustly independent factor (adjusted OR = 8.42, 95%CI: 2.53–27.98, *p* < 0.001) and significantly improved the performance of the predictive clinical model distinguished ILHP from normal lipid phenotype (AUC: 0.816, 95%CI (0.754, 0.879) vs AUC: 0.771, 95%CI (0.702, 0.840); *Z* = 2.169, *p* = 0.030). Moreover, the increased original Ct ratio of miR-221-3p to miR-222-3p in male ILHP (1.003 [0.927, 1.063] vs 0.927 [0.858, 0.967], *p* < 0.001) significantly enhanced the ability to classify male ILHP compared with the male predictive clinical model (AUC: 0.851, 95%CI (0.770, 0.933) vs AUC: 0.759, 95%CI (0.659, 0.859); *Z* = 2.474, *p* < 0.05).

**Conclusions:**

The inverted pattern of circulating miR-221-3p and miR-222-3p are potentially clinically actionable signature for molecular pathology in isolated low HDL-C phenotype.

**Electronic supplementary material:**

The online version of this article (10.1186/s12944-018-0842-1) contains supplementary material, which is available to authorized users.

## Background

MicroRNAs (miRNAs) are a class of endogenous small non-coding regulatory RNAs that play important roles in the regulation of various physiological and pathological processes [1]. Besides the intracellular biological functions, miRNAs, which can be exported or released into the extracellular space in multiple stable forms, have been proposed as innovative biomarker surrogates for human diseases [2]. Over the past few years, altered levels of miRNAs, involved in lipid and cholesterol metabolism as well as cardiovascular development and remodelling [3, 4], have been reported in plasma or serum from patients with atherosclerotic cardiovascular diseases such as acute myocardial infarction (AMI) [5] and coronary artery disease (CHD) [6]. In contrast to most candidates, cardio-specific miRNAs exhibit the robust performance in the diagnosis, prediction and prognosis of developing acute coronary syndrome (ACS) patients [7, 8]. However, clinical uses for most of circulating miRNA candidates altered in ACS patients have been not well definitely elucidated yet [9, 10]. Our previous studies showed that miR-28-5p involved in ERK2-mediated ABCA1 up-regulation did not exhibit the strong association between its increased plasma levels and higher HDL-C levels in UAP patients as expected [11]. This might be attributed to the complexity and uncertainty of established ACS patients besides its relative low levels in plasma. Some studies suggest that the changes of circulating miRNAs in cardiovascular diseases could be found easier than their intrinsic nature or interactive relationships with known or unknown factors behind combinational confounding variables [12–15]. Therefore, we are intending to investigate circulating miRNAs in asymptomatic individuals with isolated cardiovascular risk phenotype to minimize various potential confindings effects and understand their baselines in the isolated risk population.

Isolated low levels of high-density lipoprotein cholesterol (HDL-C) named isolated low HDL-C phenotype is described as a condition that coexists low HDL-C level in combination with normal LDL-C and triglyceride (TG) levels [16]. It is the most common lipid abnormality among different ethnic populations associated with increased ACS risk, especially for the Asian population [16, 17]. Individuals with isolated low HDL-C phenotype are predisposed to exhibit qualitative and quantitative traits including genetic variations [18], endothelial dysfunction [19], proinflammatory and oxidative phenotype [20], the specific composition and lipid spatial distribution of HDL particles [21], and low cholesterol efflux [22], as well as enhanced lipid peroxidation and platelet activation [23]. These changes are also observed frequently in patients with atherosclerotic diseases. However, the pattern of cardiovascular disease-related circulating miRNA candidates in isolated low HDL-C phenotype remain unknown. Decipering cardiovascular disease-derived circulating miRNA candidates in isolated low HDL-C phenotype would be useful to understand isolated phenotype-associated pathological features at circulating miRNA level.

An another key issue is how to select circulating miRNA candidates for isolated low HDL-C phenoytype. It is universally acceptable that either gender or genetic factors have major effects on HDL-C level. Based on dominant effect of gender factor on it, we firstly filtered the X-chromosome-located miRNA candidates and found the significantly increased levels of single-primary-prescursor-derived miR-221/222 mature transcripts in plasma from UAP patients (http://www.ncbi.nlm.nih.gov/geo/, accession number: GSE94605). The transcription of miR-221/222 has been reported to be controlled by estrogen receptor alpha-mediated signaling pathway [24] and ApoE-containing HDL particles [25]. Moreover, miR-222 has been reported to be transported into circulation via HDL particles [26]. Taken together these evidence, we investigated plasma miR-221/222 mature transcripts in isolated low HDL-C phenotype, and analyzed their relationships with isolated low HDL-C phenotype, gender factor and ApoE genotyping.

## Methods

### Study population

One hundred seventy-four asymptomatic subjects with isolated low HDL-C phenotype or normal lipid profiles were enrolled from Medical Health Check-up Department in Tangshan Gongren Hospital between August 2014 and November 2015. All subjects were screened according to the following criteria: 1) subjects who are less than 55 years of age; 2) subjects who were excluded if they had obesity, metabolic syndrome, diabetes, hypertension, kidney disease, and cardiovascular disease and recent medication history. Laboratory evalution included fasting Glucose, serum lipids, hepatic injury indexes. This study was approved by the Ethnics Committee of Tangshan Gongren Hospital and the written informed consent was obtained from all subjects.

### Blood samples collection and handling

Peripheral blood was drawn with EDTA-K2-anticoagulated tube and handled within 2 h. Whole blood samples were centrifuged at 3, 000 g for 10 min followed by centrifugation at 12, 000 g for 10 min to generate platelet- and cell debris-deficient plasma. The deprived plasma samples were immediately stored at − 80 °C.

### Isolation and purification of plasma miRNAs

RNA was isolated from a fixed volume (500 μL) of plasma using Ambion mirVana™ PARIS RNA isolation kit (Invitrogen) according to the manufacturer’s instructions. Extracted RNA were dissolved in a fixed volume (90 μL) of elution solution and then stored at − 80 °C.

### miRNA quantification with RT-qPCR

miRNA detection were performed with TaqMan miRNA Reverse Transcription Kit, TaqMan MiRNA Assays and TaqMan Universal PCR Master Mix (Applied Biosystems) on ABI 7500 PCR system according to the manufacturer’s protocol and modified protocol. The reaction of miRNA reverse transcription containing 1.2 μL 10 × reverse transcription (RT) buffer, a fixed volume of 8.6 μL RNA elution from the 90-μL eluate of RNA isolation as RT template, 0.1 μL 100 mM dNTPs, 0.1 μL RNase inhibitor (20 U/μL), 1.5 μL RT primer, 0.5 μL MultiScribe™ reverse transcriptase (50 U/μL) were performed following parameters: 16 °C for 60 min, 42 °C 120 min and 85 °C 5 min. The PCR reaction comprised 6 μL RT product, 10 μL of 2 × TaqMan Universal PCR Master Mix, 0.5 μL TaqMan probe (2.5 μM), and 3.5 μL DNase/RNase-free H_2_O, according to the following parameters: 95 °C 10 min followed by 50 cycles of 95 °C for 15 s, 60 for 1 min. Cycle threshold (Ct) values were determined on ABI 7500 real-time PCR system (Applied Biosystems). Mature transcripts of miR-221/222 in plasma were detected using TaqMan miRNA detection system. miR-191-5p was used as an endogenous reference for miRNA quantification as previously reported [27]. A Ct value > 40 was defined as undetectable for all miRNAs. Relative expression level was determined with the ∆Ct method and reported as 2^–∆Ct^.

### APOE genotype

Genomic DNA were extracted from whole blood samples using TIANamp blood DNA kit (TIANGEN Biotech (Beijing) Co., Ltd). The *APOE* gene has three variant alleles (ε2, ε3, ε4) which generate six different genotypes (ε2/2, ε2/3, ε2/4, ε3/3, ε3/4 and ε4/ε4). *APOE* allele carriers were defined as follows: 1) the E2 carrier includes those subjects with the ε2/ε2, ε2/ε3 or ε2/ε4 genotype; 2) the E3 carrier includes those with the ε3/ε3 genotype; and 3) the E4 carrier includes those with the ε3/ε4 or ε4/ε4 genotype. E2 has been shown to be associated with decreased LDL-C levels, whereas E4 is associated with increased CVD risk [28]. *APOE* genotyping analysis was performed using ABI PRISM® SNaPshot™ Mμltiplex Kit (Applied Biosystems) on ABI 9700 PCR system and 3730XL DNA analyzer.

### Statistical analysis

The statistic analyses were performed using GraphPAD Prism 5.0 and R bioconductor 3.0.1. Continuous variables were tested for normal distribution with the D’Agostino and Pearson ominbus normality test. Normally distributed continuous variables were presented as mean ± SD. Two groups were compared using the Student *t* test and the Mann-Whitney *U* test, as appropriate. Multiple comparisons were compared using the *Kruskal-Wallis* test, and the *P* values were corrected using the Benjamini-Hochberg (BH) method to control the false discovery rate. *Spearman* rank correlation test was performed to identify the possible relationships between circulating miRNAs and baseline characteristics of clinical laboratory parameters. Receiver operating characteristic (ROC) curves were used to evaluate the performance of univariate or multivariate logistic model. The optimal cut-off point of ROC curve was chosen at which the Youden’s index was maximal. Linear or logistic regression analysis was performed to explore the associations among circulating miRNAs, *APOE* allele carriers and isolated low HDL-C phenotype, find the optimized combination of predictive variables and build a model of predicted probability for defining isolated low HDL-C phenotype. Variables that were not normally distributed were transformed to their natural logarithm in linear regression analysis. *p* < 0.05 was considered to indicate statistical significance.

## Results

### Baseline characteristics of participants

One hundred seventy-four subjects (88 for isolated low HDL-C phenotype and 86 for normal lipid phenotype, Additional file 1: Table S1) were screened from approximately more than 400 participants from Healthy Check-up Department during August, 2014 to November, 2015. All subjects enrolled in this study shared common traits including normal non-HDL-C profiles (TC < 6.00 mmol/L, LDL-C < 3.30 mmol/L and TG < 1.70 mmol/L), fasting glucose (FG < 6.10 momol/L), biochemistry tests of liver injury (ALT and AST ≤ 40; GGT ≤ 50) and the removal of other known CVD risk factors. Isolated low HDL-C phenotype is defined as subjects with HDL-C level < 1.04 mmol/L in male or 1.16 mmol/L in female refered to phenotype-associated publication [29], especially for female. As shown in Additional file 1: Table S1, three of 15 non-HDL parameters within normal reference range had significant difference between isolated low HDL-C and normal lipid phenotype (*p* < 0.001 for TC and TG; *p* = 0.032 for ALT). Gender-based clinical features as shown in Additional file 1: Table S2. Several variables including TC, HDL-C, TG, ALT, AST, GGT, HGB, PLT and RBC had significant difference among gender-based subgroups.

### Circulating miR candidates and isolated low HDL-C phenotype

**Fig. 1:**
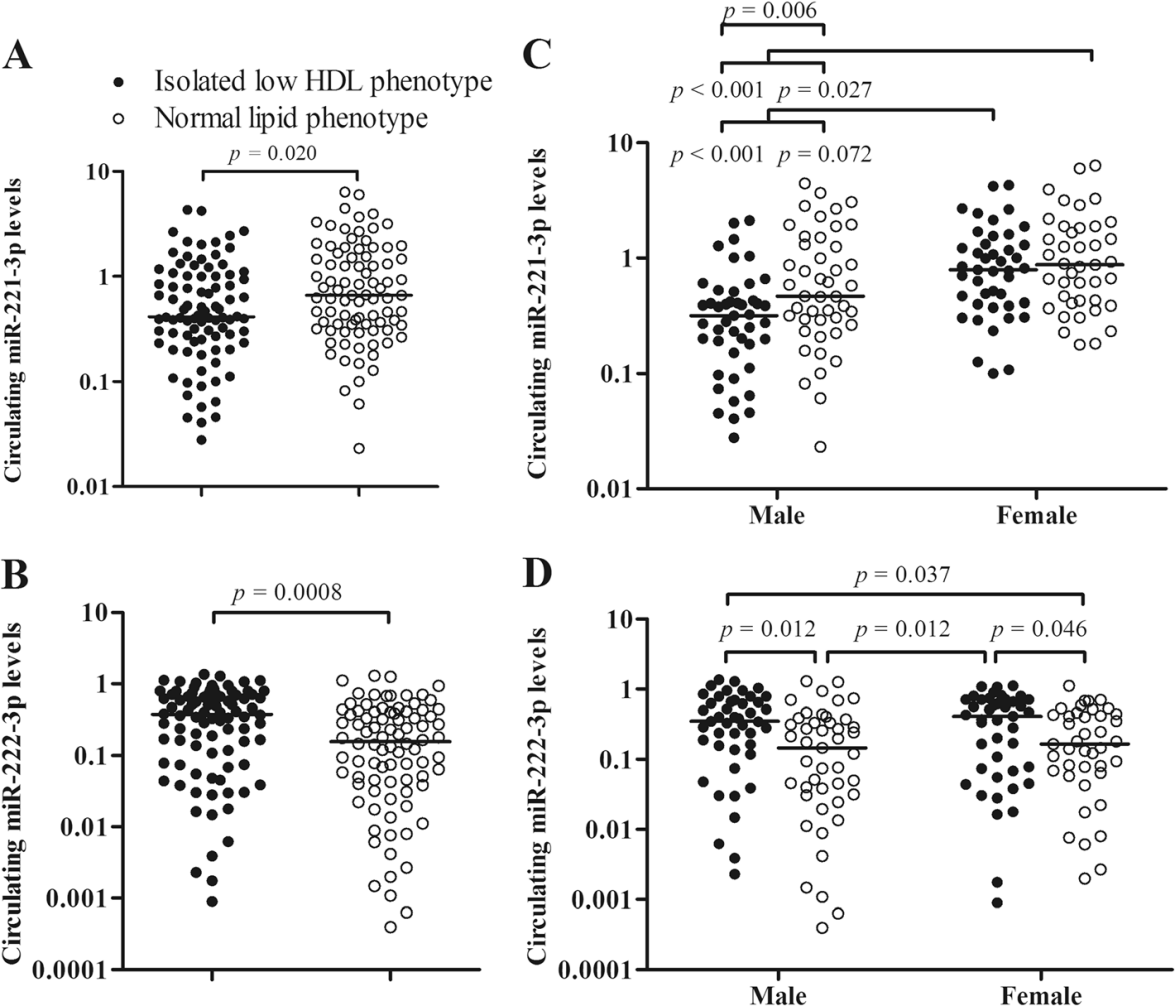
Circulating miRNAs levels in isolated low HDL-C phenotype. Circulating miR-221-3p levels (**a**) are increased in isolated low HDL-C phenotype, whereas circulating miR-222-3p levels (**b**) are decreased in isolated low HDL-C phenotype. Isolated low HDL: Isolated low HDL-C phenotype; Normal: Normal lipid phenotype. miR-221-3p levels (**c**) are significantly increased in female subjects with both isolated low HDL-C phenotype and normal lipid phenotype in comparison with those of male subgroups. **d** Circulating miR-222-3p levels are significantly increased in either male or female isolated low HDL-C phenotype compared with the corresponding normal lipid phenotypes, respectively. The *p* value is adjusted using the Benjamini-Hochberg (BH) method in multiple comparisons

Relative levels of miR-221-3p and miR-222-3p in plasma from asymptomatic subjects (*n* = 174) with normal lipid phenotype or isolated low HDL phenotype were detected using TaqMan stem-loop miRNA assay system. miR-221-3p and 222-3p as well as an endogenous miRNA, miR-191-5p were clearly detected in all of plasma samples. There were decreased levels of miR-221-3p (0.415 [0.243, 1.001] vs 0.658 [0.339, 1.574]; *p* = 0.002; Fig. 1a) and increased levels of miR-222-3p (0.379 [0.086, 0.707] vs 0.156 [0.042, 0.409]; *p* < 0.001; Fig. 1b) in isolated low HDL phenotype (*n* = 88) in comparison with normal lipid phenotype (*n* = 86). The inverted pattern of miR-221-3p and miR-222-3p in isolated low HDL-C phenotype is similar with that in morbid obesity [30].

In gender-based subgroup analysis, miR-221-3p (χ^2^ = 28.36, *p* < 0.001; Fig. 1c) and miR-222-3p (χ^2^ = 11.92, *p* = 0.01; Fig. 1d) were found to be significant difference. As shown in Fig. 1c, circulating miR-221-3p levels were predominantly elevated in female with either isolated low HDL-C phenotype or normal lipid phenotype compared with the male subgroups, which is similar to female-specific elevation in participants with metabolic syndrome [31]. Notably, increased circulating miR-221-3p levels were observed between male-categorized subgroups (0.320 [0.180, 0.428] vs 0.466 [0.293, 1.315], *p* = 0.006), rather than between female subgroups (0.790 [0.405, 1.311] vs 0.875 [0.428, 1.824], *p* = 0.288). Nevertheless, the increases of circulating miR-222-3p were predominantly observed in both of intra-gender isolated low HDL subgroups compared with the correponding normal lipid phenotype (male: 0.351 [0.158, 0.663] vs 0.147 [0.032, 0.335], *p* = 0.012; female: 0.413 [0.071, 0.712] vs 0.166 [0.069, 0.432], *p* = 0.046; Fig.1d).

### The association of baseline levels of clinical laboratory indexes and miR candidates

**Fig. 2:**
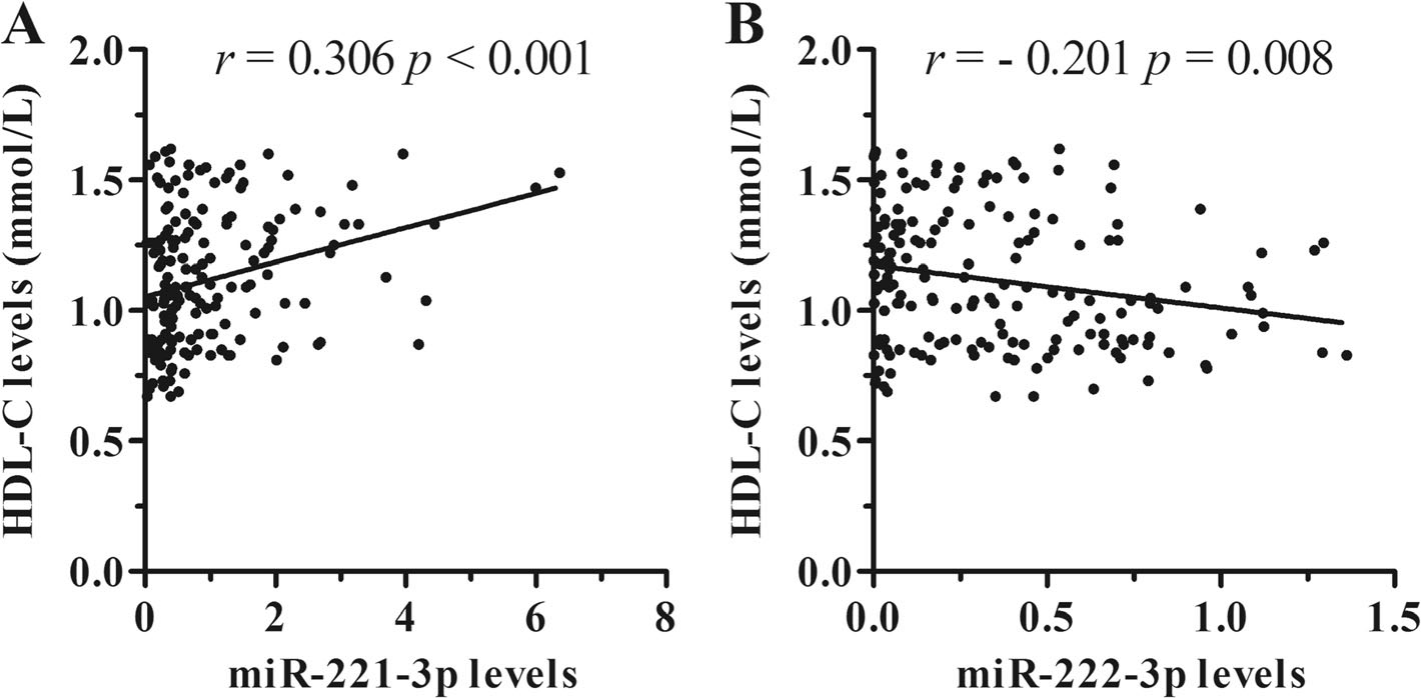
The correlations between circulating miRNA levels and serum HDL-C levels. Circulating miR-221-3p levels (**a**) are positively correlated with serum HDL-C levels, whereas circulating miR-222-3p levels (**b**) are inversely correlated with serum HDL-C levels in asymptomatic subjects (*n* = 174)

The relationships between clinical laboratory indexes and circulating miRNAs levels in asymptomatic subjects (*n* = 174) with both normal lipid phenotype and isolated low HDL-C phenotype were firstly analyzed using Spearman’s correlation analysis and linear regression analysis. Serum HDL-C levels were correlated with both of miRNA candidates (miR-221-3p: *r* = 0.306, *p* < 0.001, Fig.2a; miR-222-3p: *r* = − 0.201, *p* = 0.008, Fig.2b). The correlations with other clinical indexes were shown in Additional file 1: Table S3. Circulating miR-222-3p levels were not correlated with all the other clinical indexes (all *p* > 0.05), whereas circulating levels of miR-221-3p were correlated with 7 non-HDL-C clinical indexes, respectively (*p* < 0.05). Furthermore, step-wise linear regression analysis showed that plasma miR-222-3p levels were associated with HDL-C levels and absolute count of RBC, whereas plasma miR-221-3p were associated with five clinical laboratory indexes including gender, HDL-C, LDL-C, etc. (Additional file 1: Tables S4 and S5).

Binary logistic regression analyses were further performed to deduce the association between isolated low HDL-C phenotype and the two miRNA candidates. Our results showed that isolated low HDL-C phenotype was strongly associated with increased circulating miR-222-3p levels (adjusted OR = 8.42, 95%CI: 2.53–27.98, *p* < 0.001), rather than that of miR-221-3p (adjusted OR = 0.67, 95%CI: 0.45–1.00, *p* = 0.051; Table 1) after adjustment for baseline characteristics of non-HDL clinical laboratory parameters. These results indicate that circulating miR-222-3p level is an independent factor for isolated low HDL-C phenotype.

**Table 1:** Associations of circulating miRNA indexes with isolated low HDL-C phenotype

Models	miR-221-3p		miR-222-3p		miR-221-3p/222-3p Ct ratio	
	OR (95%CI)	*p* value	OR (95%CI)	*p* value	OR (95%CI)	*p* value
Crude	0.67 (0.49, 0.93)	0.018	5.45 (1.99, 14.88)	< 0.001	996.49 (27.34, 36,324.83)	< 0.001
Adjusted, model^a^	0.67 (0.45, 1.00)	0.051	8.42 (2.53, 27.98)	< 0.001	5617.96 (59.09, 534,118.17)	< 0.001

^a^ Model: adjusted for clinical laboratory indexes including LDL-C, TG, ALT, AST, GGT, FG, HGB, PLT, and absolute counts of WBC, RBC, neutrophil and lymphocyte, except for HDL-C. *OR* odd ratio, *CI* confidence interval

### Circulating miR-222-3p improve the performance of the predective clinical model for isolated low HDL-C phenotype

Predictive clinical model was firstly estimated using step-wise regression analysis (Additional file 1: Table S6), and then ROCs’ performance of circulating miR-222-3p on defining isolated low HDL-C phenotype was analyzed compared with the predictive clinical model. As shown in Table 2, univariate miR-222-3p yielded an AUC of 0.648 (95% CI, 0.566–0.730; *p* < 0.001). The AUC performance was significantly improved when adding miR-222-3p to the predictive clinical model (AUC: 0.816, 95%CI: 0.754, 0.879 vs (AUC: 0.771, 95%CI: 0.702–0.840; *Z* = 2.169, *p* = 0.030).

**Table 2:** The performance of circulating miR-222-3p and miR-221/.222 Ct ratio for predicting isolated low HDL-C phenotype

Models	AUC (95%CI)	*p* value	Cut-off value	Specificity	Sensitivity
miR-222-3p	0.648 (0.566, 0.730)	< 0.001	0.559	0.837	0.432
The miR-221-3p/miR-222-3p ratio	0.678 (0.598, 0.758)	< 0.001	0.562	0.767	0.568
Clinical model	0.771 (0.702, 0.840)	< 0.001	0.462	0.698	0.761
Clinical model + miR-222-3p^a^	0.816 (0.754, 0.879) ^b^	< 0.001	0.413	0.663	0.879
Clinical model + miR221-3p/miR-222-3p ratio^a^	0.813 (0.751, 0.876) ^b^	< 0.001	0.423	0.686	0.818

*AUC* area under the curve. Best threshold of Cut-off value were estimated using youden method. ^a^ The AUC, sensitivities and specificities of the combination model of miR-222-3p and clinical model was compared with that of clinical model. ^b^*p* < 0.05

### Using the Ct ratio of miR-221-3p and miR-222-3p to define male isolated low HDL-C phenotype

**Fig. 3:**
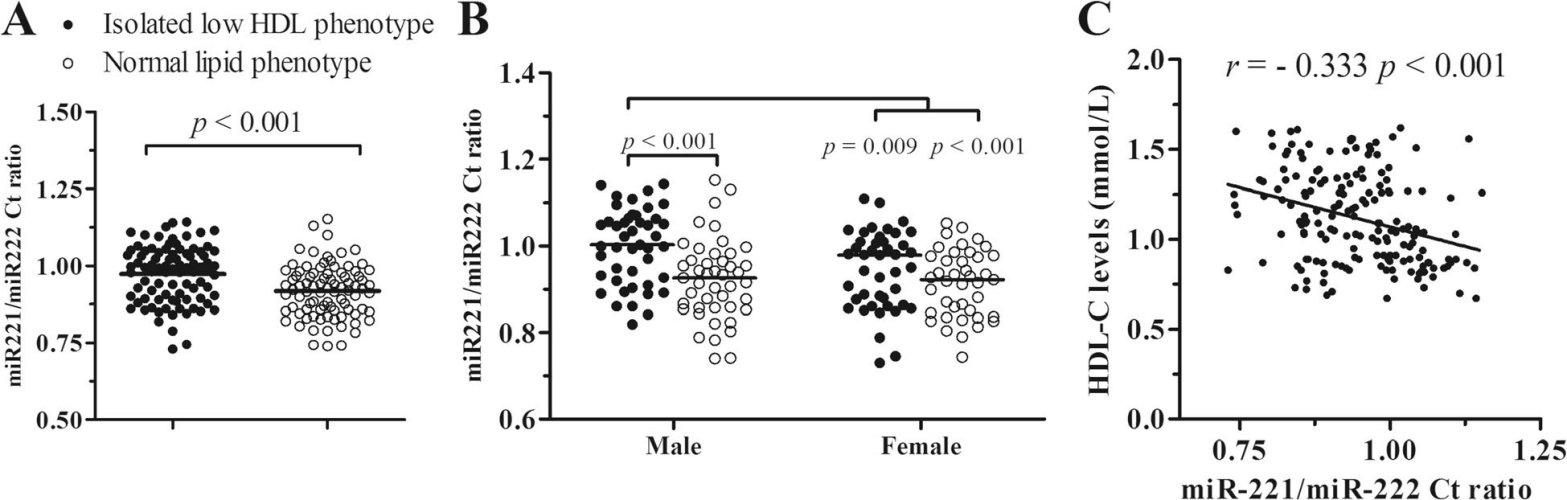
The ratio of miR-221-3p and miR-222-3p and isolated low HDL-C phenotype. **a** The original Ct ratio of circulating miR-221-3p to miR-222-3p are significantly elevated in isolated low HDL-C phenotype. **b** Compared with the corresponding normal lipid phenotype, male subjects with isolated low HDL phenotype exhibit the increased miR-221-3p/222-3p ratio, while the female isolated low HDL-C phenotype have a increased trendence. **c** The original Ct ratio of miR-221-3p to miR-222-3p are inversely correlated with serum HDL-C levels in asymptomatic subjects (*n* = 174). The *p* value is adjusted using the Benjamini-Hochberg (BH) method in multiple comparisons

According to the opposite changes of miR-221-3p and miR-222-3p in isolated low HDL-C phentype, the ratio of miR-221-3p to miR-222-3p original Ct value was analyzed. The miR-221-3p/222-3p ratios were significantly increased in isolated low HDL-C phenotype in comparison with normal lipid phenotype (0.991 [0.894, 1.044] vs 0.924 [0.855, 0.976], *p* < 0.001; Fig.3a). Compared with the corresponding normal phenotypes, male isolated low HDL phenotype exhibited significantly increased ratio (1.003 [0.927, 1.063] vs 0.927 [0.858, 0.967], *p* < 0.001), while female had an increased trendence (0.979 [0.871, 1.009] vs 0.922 [0.846, 0.976], *p* = 0.062; Fig.3b).

Correlation analysis showed that the miR-221-3p/222-3p ratios were inversely correlated with serum HDL-C levels (*r* = − 0.333, *p* < 0.001; Fig. 3c). Moreover, the substitution of the miR-221/222 ratio for circulating miR-221-3p and miR-222-3p levels showed the compromised features with the other clinical indexes (Additional file 1: Table S3).

Multivariate logistic regression analysis showed that the miR-221-3p/miR-222-3p ratio yielded an adjusted OR value of 5617.96 (95% CI: 59.09–534,118.17, *p* < 0.001, Table 1). As shown in Table 2, introducing the miR-221-3p/miR-222-3p ratio slightly improved the performance of the predictive clinical model on distinguishing isolated low HDL-C phenotype from normal lipid phenotype (*Z* = 1.972, *p* = 0.049) although there was no significant difference between the original Ct ratio and circulating miR-222-3p level (*Z* = − 0.225, *p* = 0.821). In gender-based analysis (Table 3), addition of miR-221-3p/miR-222-3p ratio (*Z* = 2.474, *p* = 0.013), instead of miR-222-3p level (*Z* = 1.844, *p* = 0.065), significantly enhanced the performance of the male predictive clinical model, while neither did miR-221-3p/miR-222-3p ratio nor circulating miR-222-3p level did improve that of the female clinical model (All *p* > 0.05).

**Table 3:** The AUCs’ performance for distinguishing subjects with isolated low HDL-C phenotype in gender-based analysis

Models	AUC (95%CI)	*p* value	Cut-off value	Specificity	Sensitivity
Male subjects					
miR-222-3p	0.671 (0.566, 0.730)	0.002	0.474	0.667	0.622
miR-221-3p/miR-222-3p ratio	0.731 (0.626, 0.836)	< 0.001	0.564	0.800	0.622
Clinical model	0.759 (0.659, 0.859)	< 0.001	0.630	0.867	0.556
Clinical model + miR-222-3p^a^	0.813 (0.721, 0.904)	< 0.001	0.522	0.800	0.778
Clinical model + miR-221-3p/miR-222-3p ratio^a^	0.851 (0.770, 0.933)^c^	< 0.001	0.430	0.756	0.889
Female subjects					
miR-222-3p	0.623 (0.501, 0.745)	0.026	0.600	0.878	0.417
miR-221-3p/miR-222-3p ratio	0.624 (0.503, 0.745)	0.025	0.566	0.780	0.512
Clinical model	0.808 (0.714, 0.901)	< 0.001	0.548	0.854	0.674
Clinical model + miR-222-3p^b^	0.847 (0.761, 0.934)	< 0.001	0.421	0.780	0.837
Clinical model + miR-221-3p/miR-222-3p ratio^b^	0.841 (0.756, 0.926)	< 0.001	0.556	0.853	0.721

*AUC* area under the curve. Best threshold of Cut-off value were estimated using youden method

^a, b^ The AUC, sensitivities and specificities of the combination model of either miR-222-3p or miR-221-3p/miR-222-3p ratio and clinical indexes were compared with that of the predictive clinical model for male and female subjects, respectively

^c^*p* < 0.05

### *APOE* genotype have no effect on circulating miR-221/222-3p

Considering *ApoE*-mediated inhibitory effect on miR-221/222 under cellular condition as well as the evaluation on whether participants were randomly enrolled in this study [25, 28], we performed *APOE* genotyping and Hardy-Weinberg equilibrium (HWE) testing. Genotypes and allele frequencies of *APOE* polymorphisms were shown in Additional file 1: Table S7. The distribution of *APOE* genotypes in normal lipid phenotype and/or isolated low HDL-C phenotype fullfilled Hardy-Weinberg equilibrium (χ^2^ = 6.18, *p* = 0.186; χ^2^ = 5.20, *p* = 0.267; χ^2^ = 9.37, *p* = 0.052; Additional file 1: Table S7), suggesting that the sampling bias problem in this study was avoided. Multinomial logistic regression analysis showed that there were no association between *APOE* allele carriers and the two miRNA candidates as well as their original Ct ratio (Additional file 1: Table S8).

## Discussion

Several studies has been attempted to explore a set of circulating miRNA candidates potential for clinical utilities on diagnosis and prevention of cardiovascular diseases [10]. Although the differentially expressed circulating miRNAs in cardiovascular diseases could be found easily, most of circulating miRNA candidates could lose statistical significance once adjusted for various risk factors such as age, gender, smoking, lipid indexes, hypertension and metabolic diseases [12–15]. It is necessary to investigate cardiovascular disease-derived miRNAs in isolated risk phenotype prior to onset of cardiovascular diseases. Herein, in this study, we performed the baseline investigation of CVD-derived circulating miR-221/222 mature transcripts as well as APOE genotyping in asymptomatic subjects to enhance our understandings on their molecular pathological features in specific risk phenotype. We found that subjects with isolated low HDL-C phenotype exhibit increased levels of miR-222-3p and decreased levels of miR-221-3p in plasma. Female subjects with either isolated low HDL-C phenotype or normal lipid phenotype exhibited higher miR-221-3p levels than male subjects. Circulating miR-222-3p levels are robustly associated with isolated low HDL-C phenotype. Moreover, the inverted pattern of circulating miR-221-3p and miR-222-3p in isolated low HDL-C phenotype provide us an opportunity to apply the original Ct ratio to annotating molecular pathology of isolated low HDL-C phenotype.

MIR-221/222 has been reported to play important roles in various physiological and pathological processes in cardiovascular system [32], suggesting that miR-221/222 may be a potential cardiovascular biomarker and a new therapeutic target in cardiovascular diseases. The levels of *miR-221/222* mature transcripts in different circulating cell-free conditions including plasma, serum or microvesicles were investigated in cardiovascular risk factors such as aging [33], endurance training [34, 35], obesity [30, 31, 36, 37], T2DM [38], stable acute coronary syndrome [6], non-ST-segment elevation myocardial infarction (NSTEMI) [39], and atherosclerosis [40, 41]. Our findings revealed the inverted pattern of decreased circulating miR-221-3p versus increased miR-222-3p levels in isolated low HDL-C phenotype in comparison with normal lipid phenotype, which is similar to the pattern of miR-221/222-3p in morbidly obese patients [30]. Notably, circulating miR-222-3p levels were robustly associated with isolated low HDL-C phenotype, whereas miR-221-3p exhibited the stronger association with gender factor than miR-222-3p. miR-221-3p and/or miR-222-3p have been shown to be associated with body mass index [31], weights [36] and endurance training [42]. It is well known that the reduced cardiovascular risk should be beneficial from raising HDL-C level, modest weight loss and aerobic exercise. These features of miR-221-3p/miR-222-3p in cell-free conditions support their roles in cardiovascular system and could become a promosing target to cardiovascular health management.

There are various types of miRNA carriers including lipoproteins, exosome and microparticles in circulation system [43]. A set of HDL-transported miRNAs such as miR-222 were found to be varied with HDL subfraction from subjects with familial hypercholesterolemia [26]. With the increase of HDL-C levels, more and more miR-222 should be transported into circulation through HDL particle. However, it is hardly ascertained whether its integrated levels in plasma sample including HDL, LDL, exosome as well as other carriers exhibt the same pattern as that in HDL particle alone. In this study, plasma miR-221-3p, rather than miR-222-3p, were increased with HDL-C levels, and this increases should be attributed to the difference between female and male. Nevertheless, subjects with higher HDL-C levels exhibited lower plasma miR-222-3p levels. The pattern of plasma miR-221-3p and miR-222-3p in isolated low HDL-C phenotype is contradictory to the mechanism on coordinated transcription of miR-221/222 through estrogen receptor alpha (ERα)-mediated inhibition in cells [24]. In addition, ApoE isoforms are another transcriptional repressor for miR-221/222 [25]. Unfortunately, ApoE allele carriers were not associated with this pattern at the genetic level. Although its mechanism remains unknown, the inverted pattern of miR-221-3p and miR-222-3p as well as the female-specific increases in miR-221-3p in cell-free conditions were also reported in patients with morbid obesity [30] or metabolic syndrome [31]. It is well known that subjects with morbid obesity or metabolic syndrome usually represent low HDL-C levels and high TG levels. Taken together these evidences, the specific pattern of extracellular miR-221/222-3p may be distinctive for increased cardiovascular risk.

There have always been several key issues such as reference selection for circulating miRNA quantification and the accuracy of single-miRNA assay in the translation of biomarkers into clinical practice. It is widely recommended to use one or a panel of invariant endogenous normalizers and/or exogenous synthetic small RNA for miRNA qPCR quantification, and relative expression levels are reported as -∆Ct or -∆∆Ct and the log2-transformed value [44]. In this study, the inverted signature of plasma miR-221-3p and miR-222-3p in isolated low HDL-C phenotype made us achieve the substitution of the original Ct ratio of miR-221-3p to miR-222-3p for classic qPCR quantification method to provide more comprehensive and accuracy information as a mirror of miR-221/222 gene at circulating plasma level for predicting isolated low HDL-C phenotype, especially in distinguishing male subjects with isolated low HDL-C phenotype type from normal lipid phenotype. Moreover, its usage overcame the uncertainty of reference selection for circulating plasma miRNAs. The method using the ratio of miR-221/222-3p in plasma sample should be at least suitable for isolated low HDL-C phenotype and morbid obesity although it could be affected by other potentially pathological conditions.

## Conclusions

The relationship between isolated low HDL-C phenotype/low HDL-C level and cardiovascular diseases has been explored in the context of Epidemiology, genetics and HDL dysfunctional. We explored the molecular pathological feature of isolated low HDL-C phenotype via profiling CVD-derived circulating miRNA candidates and found the robust association between miR-222-3p and isolated low HDL-C phenotype, and the inverted pattern of miR-221-3p/222-3p and female-specific elevation of miR-221-3p. The use of the original miR-221-3p and 222-3p Ct values exclusively exhibits the advantage over classic miRNA quantification analysis for clinical translation. Given the dominant role of miR-221/222 in vascular biology and atherosclerotic disease, the specific pattern for isolated low HDL-C phenotype combined with serum HDL-C testing may be useful in healthy management of isolated CVD risk phenotype. In the following study, we would explore whether both the inverted pattern and the original Ct ratio of miR-221/222 in cell-free circulating status are suitable for early diagnosis and prevention of major adverse cardiovascular event in isolated low HDL-C phenotype risk population and investigate their patterns in distinct isolated cardiovascular risk phenotypes including isolated high LDL-C and TG phenotypes.

## Additional file

Additional file 1: **Table S1.** Clinical Characteristics of the Study Population. **Table S2.** Gender-based Clinical Characteristics of the Study Population. **Table S3.** The correlations between circulating miRNAs and clinical laboratory indexes. **Table S4.** Stepwise linear regression analysis for plasma miR-222-3p in all subjects. **Table S5.** Stepwise linear regression analysis for plasma miR-221-3p in all subjects. **Table S6.** Clinical model predicted by stepwise logisitic analysis. **Table S7.** Genotype and allele frequencies of the *APOE* polymorphisms and HWE in this study. **Table S8.** The association between circulating miRs and *APOE* carriers. (DOC 117 kb)

## Abbreviations

ACS: Acute coronary syndrome
ALT: Alanine aminotransferase
AST: Aspartate aminotransferase
CVD: Cardiovascular disease
FG: Fasting glucose
GGT: Gamma-glutamyl transferase
HDL: High-density lipoprotein
HGB: Hemoglobin
HWE: Hardy-Weinberg equilibrium
ILHP: Isolated low HDL-C phenotype
LDL: Low-density lipoprotein
LYM: Lymphocyte
miRNAs: MicroRNAs
NEU: Neutrophill
NSTEMI: Non-ST-segment elevation myocardial infarction
PLT: Platelets
RBC: Red blood cell
TC: Total cholesterol
TG: Triglyceride
UAP: Unstable angina pectoris
WBC: White blood cell
