# Supplementary material for: The inverted pattern of circulating miR-221-3p and miR-222-3p associated with isolated low HDL-C phenotype
Source: Lipids Health Dis. 2018 Aug 16;17:188. doi: 10.1186/s12944-018-0842-1 (PMC6097213; doi:10.1186/s12944-018-0842-1)
Supplement: Supplementary file 1 — Table S1. Clinical Characteristics of the Study Population. Table S2. Gender-based Clinical Characteristics of the Study Population. Table S3. The correlations between circulating miRNAs and clinical laboratory indexes. Table S4. Stepwise linear regression analysis for plasma miR-222-3p in all subjects. Table S5. Stepwise linear regression analysis for plasma miR-221-3p in all subjects. Table S6. Clinical model predicted by stepwise logisitic analysis. Table S7. Genotype and allele frequencies of the APOE polymorphisms and HWE in this study. Table S8. The association between circulating miRs and APOE carriers. (DOC 117 kb) [file 12944_2018_842_MOESM1_ESM.doc]

**Table S1. Clinical Characteristics of the Study Population**

| Characterisitics | | asymptomatic subjects ( n = 174) | | *P* value |
| --- | --- | --- | --- | --- |
|  |  | Normal lipid phenotype | Isolated low HDL-C phenotype |  |
|  | Age (yrs) | 36 [30, 44] | 33 [28, 42] | 0.078 |
|  | Gender (F/M) | 41/45 | 43/45 | 0.881 |
|  | TC (mmol/L) | 4.62 ± 0.44 | 4.11 ± 0.46 | < 0.001 |
|  | HDL-C (mmol/L) | 1.32 [1.22, 1.48] | 0.91 ± 0.11 | < 0.001 |
|  | LDL-C (mmol/L) | 2.56 ± 0.33 | 2.59 [2.30, 2.82] | 0.935 |
|  | TG (mmol/L) | 0.88 ± 0.28 | 1.12 [0.89, 1.39] | < 0.001 |
|  | FG (mmol/L) | 4.84 ± 0.42 | 4.93 ± 0.44 | 0.180 |
|  | ALT (U/L) | 14.50 [10, 20] | 17.50 [12.25, 24.00] | 0.032 |
|  | AST (U/L) | 16 [14, 19] | 16.48 ± 3.52 | 0.679 |
|  | GGT (U/L) | 16.00 [13.00, 21.25] | 16.50 [14.00, 25.00] | 0.282 |
|  | HGB (g/L) | 143.00 [132.00, 156.30] | 144.00 ± 10.93 | 0.777 |
|  | PLT (10^9^/L) | 243.50 [216.50, 271.30] | 232.50 [202.50, 270.80] | 0.548 |
|  | WBC (10^9^/L) | 6.56 ± 1.43 | 6.81 ± 1.32 | 0.173 |
|  | RBC (10^12^/L) | 4.75 ± 0.42 | 4.81 ± 0.42 | 0.378 |
|  | NEU (10^9^/L) | 3.53 [3.53, 4.15] | 3.69 [3.14, 4.47] | 0.196 |
|  | LYM (10^9^/L) | 2.30 [1.90, 2.60] | 2.31 ± 0.52 | 0.718 |

TC, total cholesterol; HDL, high-density lipoprotein; LDL, low-density lipoprotein; TG, triglyceride; FG, fasting glucose, ALT, Alanine aminotransferase; AST, aspartate aminotransferase; GGT, Gamma-glutamyl transferase; HGB, hemoglobin; PLT, platelets; WBC, white blood cell; RBC, red blood cell; NEU, neutrophill; LYM, lymphocyte. Comparisons between isolated low HDL-C phenotype and normal lipid phenotype were performed with student’s *t* test or Mann-Whitney *U* test for continuous variables and with the Fischer exact test for categorical variables.

**Table S2. Gender-based Clinical Characteristics of the Study Population**

| Characteristics | asymptomatic subjects ( n = 174) | | | |  |
| --- | --- | --- | --- | --- | --- |
|  | Female | | Male | | *P* value |
|  | Normal lipid phenotype  (n = 41) | Isolated low HDL-C phenotype  (n = 43) | Normal lipid phenotype  (n = 45) | Isolated low HDL-C phenotype  (n = 45) |  |
| Age (yrs) | 35.71 ± 7.33 | 34.93 ± 7.06 | 37.76 ± 8.54 | 34.44 ± 8.22 | 0.202 |
| TC (mmol/L) | 4.72 ± 0.44 | 4.15 ± 0.42 | 4.53 ± 0.42 | 4.08 ± 0.49 | < 0.001 |
| HDL-C (mmol/L) | 1.47[1.27, 1.53] | 1.01[0.89, 1.05] | 1.26 ± 0.14 | 0.84 ± 0.09 | < 0.001 |
| LDL-C (mmol/L) | 2.47 ± 0.33 | 2.56[2.31, 2.83] | 2.63 ± 0.31 | 2.53 ± 0.36 | 0.170 |
| TG (mmol/L) | 0.81 ± 0.25 | 1.09 ± 0.31 | 0.94 ± 0.29 | 1.17 ± 0.31 | < 0.001 |
| FG (mmol/L) | 4.74 ± 0.39 | 4.95 ± 0.37 | 4.94 ± 0.43 | 4.91 ± 0.50 | 0.083 |
| ALT (U/L) | 11.00[9.00, 15.00] | 13.00[10.00, 17.50] | 19.13 ± 7.52 | 22.69 ± 7.96 | < 0.001 |
| AST (U/L) | 15.44 ± 3.01 | 15.00[13.00, 16.00] | 18.00[15.00, 20.00] | 17.73 ± 3.21 | < 0.001 |
| GGT (U/L) | 13.00[12.00, 16.00] | 14.00[12.00, 16.00] | 22.11±7.58 | 23.04±7.01 | < 0.001 |
| HGB (g/L) | 130.70 ± 8.81 | 133.00[128.50, 138.50] | 155.00 ± 9.54 | 155.10 ± 6.93 | < 0.001 |
| PLT (10^9^/L) | 246.70 ± 48.86 | 256.50 ± 55.77 | 232.00[217.00, 259.00] | 218.00[199.00, 250.00] | 0.030 |
| WBC (10^9^/L) | 6.50 ± 1.50 | 6.75 ± 1.31 | 6.53[5.71, 7.27] | 6.86 ± 1.34 | 0.530 |
| RBC (10^12^/L) | 4.42 ± 0.24 | 4.50 ± 0.29 | 5.05 ± 0.32 | 5.10 ± 0.29 | < 0.001 |
| NEU (10^9^/L) | 3.80 ± 1.21 | 3.89 ± 0.98 | 3.61[3.01, 3.99] | 3.69[3.20, 4.47] | 0.490 |
| LYM (10^9^/L) | 2.17 ± 0.48 | 2.31 ± 0.58 | 2.34[1.99, 2.74] | 2.31 ± 0.45 | 0.298 |

TC, total cholesterol; HDL, high-density lipoprotein; LDL, low-density lipoprotein; TG, triglyceride; FG, fasting glucose, ALT, Alanine aminotransferase; AST, aspartate aminotransferase; GGT, Gamma-glutamyl transferase; HGB, hemoglobin; PLT, platelets; WBC, white blood cell; RBC, red blood cell; NEU, neutrophill; LYM, lymphocyte. Multiple comparisons were compared using the *Kruskal-Wallis* test or ANOVA as appropriate.

**Table S3**. The correlations between circulating miRNAs and clinical laboratory indexes

| Correlation coefficient | Age | HDL-C | LDL-C | TC | TG | ALT | AST | GGT | FG | HGB | WBC | RBC | PLT | NEU | LYM |
| --- | --- | --- | --- | --- | --- | --- | --- | --- | --- | --- | --- | --- | --- | --- | --- |
| miR221-3p levels | -0.038 | **0.306***** | 0.113 | **0.199**** | -0.110 | **-0.195**** | -0.068 | **-0.320***** | -0.016 | **-0.328***** | -0.012 | **-0.329***** | **0.229**** | -0.064 | 0.092 |
| miR222-3p levels | 0.049 | **-0.201***** | 0.005 | -0.095 | 0.063 | -0.081 | -0.042 | 0.011 | 0.006 | -0.102 | -0.096 | -0.084 | -0.026 | -0.062 | -0.103 |
| miR221-3p/miR-222-3p Ct ratio | 0.055 | **-0.333***** | -0.047 | **-0.177*** | 0.115 | 0.046 | 0.004 | **0.192*** | 0.024 | 0.085 | -0.043 | 0.101 | -0.131 | 0.013 | -0.123 |

Note: Clinical laboratory indexes included lipid profiles (HDL-C, TC, TG and LDL-C), hepatic injury indexes (ALT, AST, and GGT) as well as fasting glucose (FG), hemoglobin (HGB) and peripheral blood cell absolute counts including white blood cell (WBC), red blood cell (RBC), Neutrophil and lymphocyte, and platelets (PLT).

*** *P* < 0.001; ** *P* < 0.01; * *P* < 0.05.

**Table S4. Stepwise linear regression analysis for plasma miR-222-3p in all subjects**

| Indexes | Coeff./SE | *t* value | *P* value |
| --- | --- | --- | --- |
| HDL-C (mmol/L) | - 1.715 ± 0.591 | - 2.901 | 0.004 |
| RBC (10^12^/L) | - 3.871 ± 1.533 | - 2.525 | 0.012 |

The predicted variables in model were estimated based on the lowest AIC value and their strong assoications with plasma miR-222-3p levels using step-wise linear regression analysis. Variables that were not normally distributed were transformed to their natural logarithm. Coeff./SE, the coefficient/standard error of the coefficient in linear regression model. HDL, high-density lipoprotein; RBC, red blood cell.

**Table S5. Stepwise linear regression analysis for plasma miR-221-3p in all subjects**

| Indexes | Coeff./SE | *t* value | *P* value |
| --- | --- | --- | --- |
| Male vs Female | - 0.470 ± 0.179 | - 2.630 | 0.009 |
| HDL-C (mmol/L) | 0.994 ± 0.342 | 2.906 | 0.004 |
| LDL-C (mmol/L) | 1.323 ± 0.567 | 2.335 | 0.021 |
| GGT (U/L) | - 0.726 ± 0.258 | - 2.817 | 0.005 |
| WBC (10^9^/L) | 2.678 ± 0.889 | 3.013 | 0.003 |
| NEU (10^9^/L) | - 2.166 ± 0.697 | - 3.106 | 0.002 |

The predicted variables in model were estimated based on the lowest AIC value and their strong assoications with plasma miR-221-3p levels using step-wise linear regression analysis. Variables that were not normally distributed were transformed to their natural logarithm. Coeff./SE, the coefficient/standard error of the coefficient in linear regression model. HDL, high-density lipoprotein; LDL, low-density lipoprotein; GGT, Gamma-glutamyl transferase; WBC, white blood cell; NEU, neutrophill.

**Table S6. Clinical model predicted by stepwise logisitic analysis**

| Variables | Coeff./SE | Adj.OR(95%CI) | Z value | *p* value |
| --- | --- | --- | --- | --- |
| TG | 3.14/1.14 | 23.08 (6.46, 82.39) | 0.499 | < 0.001 |
| Age | - 0.05/0.02 | 0.95 (0.91, 1.00) | - 2.147 | 0.032 |
| AST | - 0.20/0.07 | 0.81 (0.71, 0.94) | - 2.840 | 0.005 |
| ALT | 0.09/0.03 | 1.09 (1.02, 1.16) | 2.682 | 0.007 |

The predicted variables of clinical model was estimated based on the lowest AIC value and their strong assoications with isolated low HDL-C phenotype using stepwise forward and backward logistic regression analysis. AIC: Akaike information criterion. Coeff./SE, the coefficient/standard error of the coefficient in logit model; OR, odd ratio; CI, confidence interval.

**Table S7. Genotype and allele frequencies of the *APOE* polymorphisms and HWE in this study**

| Groups (n) | Genotype [n(%)] | | | | | Allele frequency [n(%)] | | | HWE | |
| --- | --- | --- | --- | --- | --- | --- | --- | --- | --- | --- |
|  | ε2/ε3 | ε2/ε4 | ε3/ε3 | ε3/ε4 | ε4/ε4 | ε2 | ε3 | ε4 | χ2 | *p* |
| Normal lipid phenotype (86) | 21(23.91) | 4(4.35) | 54(61.96) | 7(8.70) | 0(1.09) | 25(14.53) | 136(79.07) | 11(6.40) | 6.18 | 0.186 |
| Isolated low HDL-C phenotype (88) | 9(9.76) | 3(3.66) | 65(75.61) | 10(10.98) | 1(0.00) | 12(6.82) | 149(84.66) | 15(8.52) | 5.20 | 0.267 |
| Total (174) | 30(17.24) | 7(4.02) | 119(68.97) | 17(9.20) | 1(0.57) | 37(10.63) | 119(81.90) | 26(7.47) | 9.37 | 0.052 |

HWE: Hardy-Weinberg equilibrium.

**Table S8. The association between circulating miRs and *APOE*** carriers

|  | E2 carrier | | |  | E4 carrier | | |
| --- | --- | --- | --- | --- | --- | --- | --- |
|  | Coeff. / SE | RRR (95%CI) | p value |  | Coeff. / SE | adj. RRR (95%CI) | p value |
| With circulating miR-221-3p levels | | | | | | | |
| Crude | -0.37/0.274 | 0.69(0.4, 1.18) | 0.172 |  | 0.2/0.172 | 1.22(0.87, 1.72) | 0.239 |
| Adjusted, model^a^ | -0.39/0.32 | 0.68(0.36, 1.27) | 0.225 |  | 0.13/0.205 | 1.13(0.76, 1.69) | 0.540 |
| With circulating miR-222-3p levels | | | | | | | |
| Crude | 0.06/0.63 | 1.07(0.31, 3.67) | 0.920 |  | 0.97/0.616 | 2.64(0.79, 8.84) | 0.115 |
| Adjusted, model^a^ | 0.31/0.729 | 1.37(0.33, 5.71) | 0.667 |  | 1.25/0.716 | 3.47(0.85, 14.14) | 0.082 |
| With the miR-221/222-3p Ct ratio | | | | | | | |
| Crude | 0.18/2.209 | 1.19(0.02, 90.68) | 0.936 |  | 0.72/2.381 | 2.06(0.02, 219.02) | 0.762 |
| Adjusted, model^a^ | 0.74/2.352 | 2.1(0.02, 211.1) | 0.752 |  | 1.79/2.423 | 5.99(0.05, 691.28) | 0.460 |

^a^ Model: adjusted for clinical laboratory indexes including LDL-C, TG, ALT, AST, GGT, FG, HGB, PLT, and absolute counts of WBC, RBC, neutrophiles and lymphocytes, except for HDL-C. Coeff./SE, the coefficient/standard error of the coefficient in logit model; RRR, relative risk ratio; CI, confidence interval.
